# Extraction of 3D distribution of potato plant CWSI based on thermal infrared image and binocular stereovision system

**DOI:** 10.3389/fpls.2022.1104390

**Published:** 2023-01-24

**Authors:** Liuyang Wang, Yanlong Miao, Yuxiao Han, Han Li, Man Zhang, Cheng Peng

**Affiliations:** ^1^ Key Laboratory of Agricultural Information Acquisition Technology, Ministry of Agriculture and Rural Affairs, China Agricultural University, Beijing, China; ^2^ Key Laboratory of Smart Agriculture System Integration Research, Ministry of Education, China Agricultural University, Beijing, China

**Keywords:** water stress, automation, photogrammetry, image registration, 3D distribution

## Abstract

As the largest component of crops, water has an important impact on the growth and development of crops. Timely, rapid, continuous, and non-destructive detection of crop water stress status is crucial for crop water-saving irrigation, production, and breeding. Indices based on leaf or canopy temperature acquired by thermal imaging are widely used for crop water stress diagnosis. However, most studies fail to achieve high-throughput, continuous water stress detection and mostly focus on two-dimension measurements. This study developed a low-cost three-dimension (3D) motion robotic system, which is equipped with a designed 3D imaging system to automatically collect potato plant data, including thermal and binocular RGB data. A method is developed to obtain 3D plant fusion point cloud with depth, temperature, and RGB color information using the acquired thermal and binocular RGB data. Firstly, the developed system is used to automatically collect the data of the potato plants in the scene. Secondly, the collected data was processed, and the green canopy was extracted from the color image, which is convenient for the speeded-up robust features algorithm to detect more effective matching features. Photogrammetry combined with structural similarity index was applied to calculate the optimal homography transform matrix between thermal and color images and used for image registration. Thirdly, based on the registration of the two images, 3D reconstruction was carried out using binocular stereo vision technology to generate the original 3D point cloud with temperature information. The original 3D point cloud data were further processed through canopy extraction, denoising, and k-means based temperature clustering steps to optimize the data. Finally, the crop water stress index (CWSI) of each point and average CWSI in the canopy were calculated, and its daily variation and influencing factors were analyzed in combination with environmental parameters. The developed system and the proposed method can effectively detect the water stress status of potato plants in 3D, which can provide support for analyzing the differences in the three-dimensional distribution and spatial and temporal variation patterns of CWSI in potato.

## 1 Introduction

Global climate change and water scarcity lead to a severe negative impact on crop yield. Among the increasing research on crop precision irrigation, water stress detection has attracted increasing attention. Potato is the fourth most important food crop in the world after wheat, rice, and maize (FAOSTAT, 2020). Due to the relatively shallow root-zone depth coupled with the low to medium soil field capacity of the coarse-textured soils commonly used for their cultivation (Rud et al., 2014), the potato plant has a high sensitivity to water stress, which affects its growth and, in turn, its yield and quality. Therefore, it is crucial to improve potato yield and quality by effectively water stress status monitoring.

The immediate response of crop to water stress is to close leaf stomata, resulting in increasing canopy temperature. Stomatal conductance is a vital indicator of plant water stress, and canopy temperature is a surrogate indicator for stomatal conductance (Prashar and Jones, 2016). Idso et al. (1981) proposed the crop water stress index (CWSI) based on crop canopy temperature, which has been proved to effectively reflect the water stress status of the crop since it was proposed. This indicator can be calculated by three methods: empirical approach (Idso et al., 1981), analytical approach (Jackson et al., 1981), and direct approach (Jones, 1999). The CWSI calculated by the three methods can be abbreviated as CWSI_e_, CWSI_a_, and CWSI_d_, respectively (Maes and Steppe, 2012). Among them, CWSI_e_ and CWSI_a_ rely on meteorological information such as ambient temperature, humidity, wind speed, etc. In the measurement process, CWSI_d_ only needs a thermal infrared image to simultaneously acquire the temperatures of the dry and wet reference surfaces (T_dry_ and T_wet_), and the temperature of crop canopy or leaf (T_c_). T_dry_ represents the temperature of a non-transpiring leaf with completely closed stomata, and T_wet_ represents the leaf temperature when stomata are fully open (undisturbed transpiring leaf). Due to the application of artificial reference surfaces (Poirier-Pocovi et al., 2020), the measurement based on thermal infrared images is further simplified. Moreover, the CWSI_d_ index showed a good correlation with stomatal conductance (Maes et al., 2016), leaf water potential (Rud et al., 2014), and stem water potential (Garcia-Tejero et al., 2017).

With the development of thermal imaging technology, especially the decrease in high-performance online thermal camera price, thermal infrared image has become increasingly widely used in the agricultural field (Qiu et al., 2018). CWSI (CWSI_d_) calculation using the direct approach has become widely used. In addition to non-destructive temperature measurement, thermal infrared images can also be obtained continuously, online, and rapidly in high-resolution. Therefore, compared with other indicators such as stomatal conductance, leaf water potential, and stem water potential, the CWSI calculation using the direct approach has the potential for high-throughput water stress detection, and can be applied to precise irrigation planning and drought resistance breeding (Prashar et al., 2013). Thermal image captured by thermal imaging equipment usually contains canopy temperature and background temperature. It is a vital issue to eliminate the background noise of thermal images. One method is to separate the canopy based on the temperature difference between the canopy and background. For example, Obidiegwu et al. (2015) assessed water stress by extracting the crop canopy in thermal images around noon under solar illumination. Because the pixel resolution of the thermal camera is very low, and a single pixel can detect thermal radiation from soil and leaf, a threshold based on temperature alone may create a high degree of uncertainty in estimating Tc. The other method is to collect thermal and color images of the canopy simultaneously for alignment and geometric registration, and then the canopy area can be extracted based on segmentation algorithms of color image processing (Amogi et al., 2020; Cucho-Padin et al., 2020; Elsherbiny et al., 2021). This method requires pre-calibration based on multiple sets of images from the thermal camera and color camera, to determine the horizontal and vertical displacement vectors between the two cameras. Manual selection of the relevant control points in the two images is often needed. Gan et al. (2018) proposed a photogrammetry-based multi-modal image registration method, which achieved an average accuracy of 3 pixels on citrus canopy images.

With the development of technology, high-throughput phenotyping methods using advanced sensors and robotic platforms have shown increasing efficiency over traditional manual phenotyping methods. Studies have shown that high-throughput phenotyping techniques have achieved good results in detecting and monitoring plant health, water and nutritional status using multi-sensor data (Pereyra-Irujo et al., 2012; Kipp et al., 2014). There are many high-throughput phenotyping platforms developed by organizations and institutions which are in use today (e.g., Scanalyzer Discovery platform, LemnaTec, Germany; Phenomobile, High Resolution Plant Phenomics Centre, Australia). However, these commercial platforms are expensive and unsuitable for large-scale deployment. Therefore, there is a need to develop a low-cost and lightweight system that can meet specific crop phenotyping needs. Zhang et al. (2016) developed a three-dimension (3D) motion robotic system for automated high-throughput phenotyping of cereal crops, which can extract 20 features from data acquired by onboard thermal and multispectral cameras. Precision irrigation and drought resistance breeding also require the large-scale automatic collection of crop water stress data.

The current methods for crop CWSI calculation is often based on temperature of random canopy parts or the entire canopy, obtained on two-dimension (2D) thermal image. However, it fails to verify whether the water stress status of crops is affected by different leaf positions or different detection positions. Studies have shown that for potato plants, there are differences in different leaf positions of plants due to the transferability of chlorophyll. For example, Sun et al. (2018) took Atlantic cultivars at the flowering stage as the research object, drew a visual distribution map of chlorophyll in isolated potato leaves at different leaf positions, and found that the chlorophyll content increased from bottom to top. Also, Sun et al. (2019) analyzed the two-dimensional distribution of water content in isolated potato leaves by hyperspectral imaging, and found that water stress increased, and the leaves started to lose water from the edge and gradually spread to the middle of the leaves. The above research shows that the water stress status of potato plants may be affected by the leaf position and detection position, which reflects the necessity of studying the differences of potato CWSI in the 3D distribution. In general, there are many ways to acquire 3D point cloud data (PCD) of a plant. It has been reported that Narvaez et al. (2016) obtained thermal distribution of pear trees in 3D using LiDAR and thermal camera. However, the price of LiDAR is generally high. Rossi et al. (2022) proposed an algorithm to automatically collect plant structural parameters based on a phenotyping platform and structure-from-motion (SFM) method, and applied the algorithm to monitor the dynamic response of the plant to early water stress. The SFM is an offline algorithm for 3D reconstruction of a series of disordered images, which limits its commercial use. 3D reconstruction based on stereo vision technology, an image-based 3D information acquisition method, has low cost and simple equipment, and is one of the most commonly used reconstruction methods. Laguela et al. (2012) used image matching and fusion techniques to combine thermal imaging and metric information to acquire 3D thermal models. Yang et al. (2018) developed an imaging system consisting of two smartphones and a low-cost thermal infrared camera, and the images captured by it were fused for 3D thermal model reconstruction.

This study aims to extract 3D distribution of potato plant CWSI at low cost using a thermal and a binocular camera. Firstly, a 3D motion robotic system integrated with a 3D thermal imaging system was developed for automated high throughput acquisition of potato plant thermal image, binocular images (a pair of color images), and temperature data. Then, specific methods for generating a 3D thermal model of the potato plant canopy were developed. The objectives of this study are to: (1) develop a low-cost 3D platform and an image acquisition control system, which has the function of positioning the image acquisition module at predefined position and triggering the control system to acquire images; (2) propose a method for fast pixel-level registration of thermal and color images, and (3) acquire the 3D CWSI distributions of the potato plant, and analyze its variation characteristics and influencing factors in time series.

## 2 Material and methods

### 2.1 3D motion robotic system

**Figure 1:**
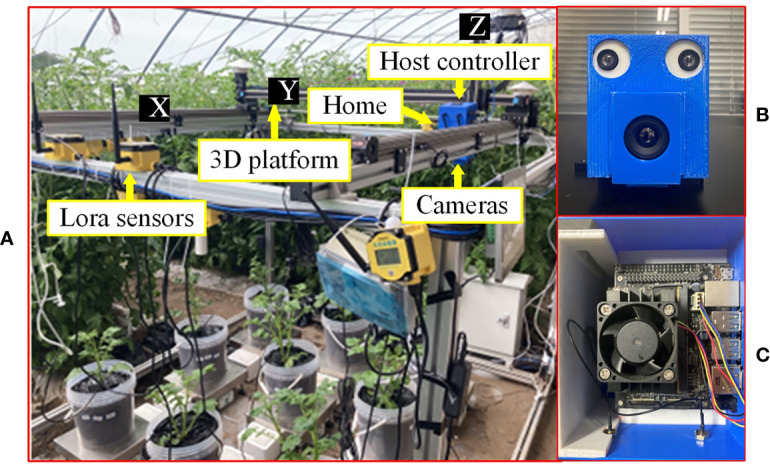
Equipment and apparatus used for experiments in this study: **(A)** overall experimental platform, **(B)** image acquisition module, and **(C)** host controller.

As shown in **Figure 1A**, the hardware of the 3D motion robotic system adopted a modular design, consisting of a 3D platform, an image acquisition module, and a host controller.

The 3D platform consists of an XYZ three-axis gantry aluminum frame, three stepper drive control integrated motors, and a single-chip microcomputer. The platform was designed as 1.8 m×1.8 m×1.5 m (L×W×H) in dimension. The sliders are driven by motors to move along the three axes. The single-chip microcomputer is a development board integrated with the STM32F103RCT6 (ST, Geneva, Switzerland) chip, which sends control signals to the motor. The maximum payload of the Z-axis for carrying the image acquisition module is 10 kg. The “Home” position or coordinate origin of the 3D platform is at one of the corners of the platform (**Figure 1A**).

The image acquisition module consists of a thermal camera (Wuhan Guide Infrared Co., Ltd, Wuhan, China) with a model of IPT384 and a binocular USB camera (Pixel XYZ, Wuhan, China). The thermal camera can capture thermal pseudo-color images with a resolution of 384×288 pixels, and save the temperature of each pixel (measurement resolution is 0.1°C) into a text file. The baseline of this binocular USB3.0 camera is 60 mm, and the left and right cameras can both capture color images in 1280×720-pixel resolution. As shown in **Figure 1A**, the image acquisition module was fixed on the slider of the Z-axis (indicated by Z in **Figure 1A**) of the 3D platform. For ease of installation, a camera frame was designed and 3D printed to mount the two cameras (**Figure 1B**).

The host controller is a Jetson Nano (NVIDIA Corporation, California, USA) running Ubuntu 18.04 system. The host controller sends commands to the single-chip microcomputer through the interface to control the 3D platform to move in three directions. Furthermore, the host controller controls the capturing of images of crop canopy by the cameras through communications with the image acquisition module. As shown in **Figure 1A**, the host controller and power module were fixed above the image acquisition module. To facilitate installation, a frame was designed and 3D printed to mount the host controller (**Figure 1C**).

**Figure 2:**
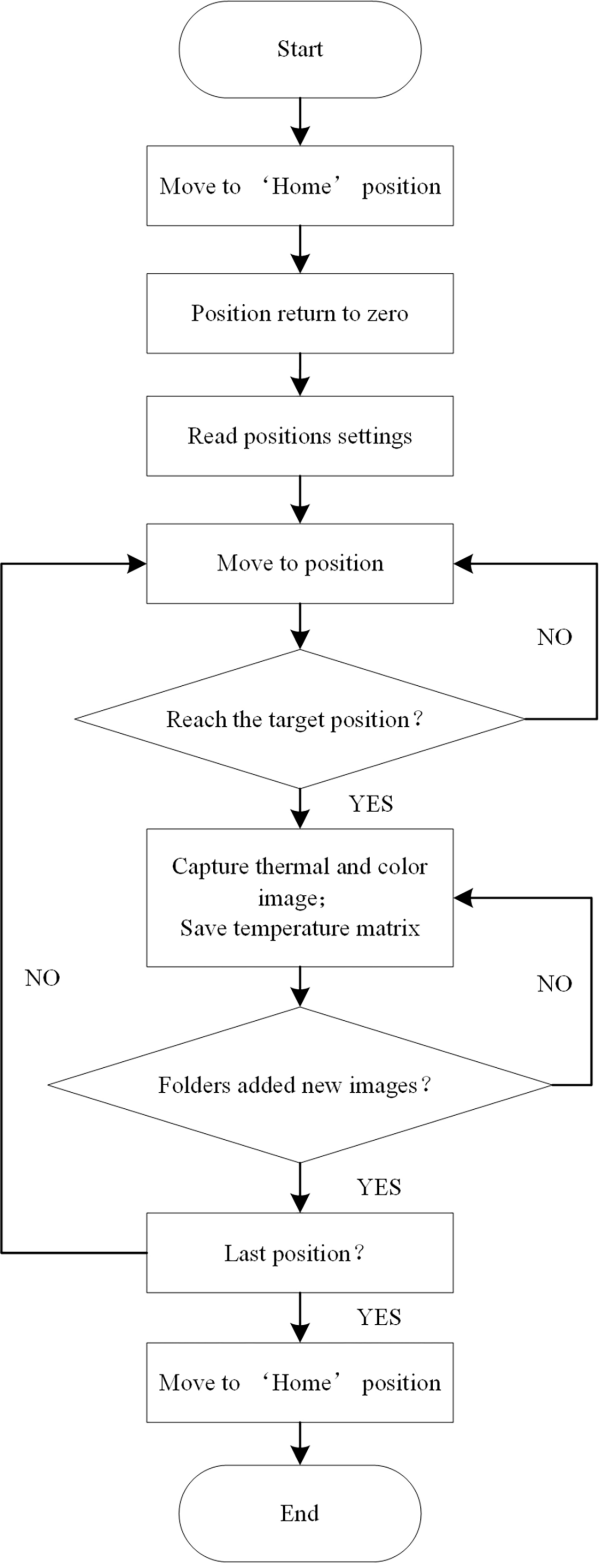
Workflow chart of the 3D robot system.

The host controller uses the Robot Operating System (ROS) software architecture to write each functional module in the form of nodes, which are divided into “AxisMoveNode” (AMN), “IrCaramIpt384NodeV2016” (IrCN), and “RGBCaramNode” (RGBCN). Communication between the nodes is implemented in the form of publish/subscribe messages. The workflow of the 3D motion robotic system is shown in **Figure 2**. In the initial stage, the AMN controls the motors to drive the sliders to move to the origin position and return the coordinates to zero. Then, the AMN controls the motor to drive the slider to move to the preset target position, and judges whether the slider has reached the target position through the position coordinate information fed back in real-time. After reaching the target position, the IrCN and the RGBCN receive the message of reaching the target position published by the AMN, and then control the image acquisition module to capture the images of the crop canopy and the temperature data to the local folder, and publish the status message of the folder at the same time. The AMN determines whether to go to the next target location by judging whether all images and temperature data are newly added to the folders. Until all the target positions are traversed, the AMN controls the motors to drive the sliders to move to the origin position. Hence, a round of inspection is completed.

### 2.2 Data collection

In the period from April 24th to May 1st (7 days), 2022, a water stress experiment on potato plants (Netherlands 15) was conducted in the No.9 greenhouse facilities of the National Precision Agriculture Research Base, Beijing, China (40◦18′N, 116◦45′E). Potatoes are grown in pots, with peat and coconut bran (at a volume ratio of 3:1) as the substrate, covered with a black plastic. There were two experimental groups (control and treatment groups, each has four potato plants), and the two groups were treated with the same irrigation from planting to the beginning of the experiment. After the first day of the experiment, the control group was fully irrigated, and the treatment group was not irrigated (Gerhards et al., 2016).

In this study, a wet reference surface was built following the steps proposed by Meron et al. (2013). The CWSI can be calculated by function (1) (Jones et al., 2002).


(1) $$\text{ CWSI= }\frac{{\text{T}}_{\text{c}}\;{\text{- T}}_{\text{wet}}}{{\text{T}}_{\text{dry}}\;{\text{- T}}_{\text{wet}}\;}$$

where *T_c_*, *T_wet_*, and *T_dry_* are the potato plant canopy temperature, dry and wet reference surface temperatures, respectively. *T_dry_* was replaced by air temperature *T_air_* plus 7°C (Rud et al., 2014). The CWSI values are in the range of 0-1, and the larger the value, the greater the water shortage pressure.

In this experiment, the cameras acquired plant data perpendicular to the ground at a height of approximately 1.2m to 1.5m every day. The next day, the collection height were readjusted according to the natural growth of the potato to ensure that the entire canopy is included in the image as much as possible. When collecting thermal images, *T_air_* and illumination were measured by LoRa sensors (IntelliFuture, Hebei, China). The real-time *T_air_* and illumination were uploaded to the cloud platform through the LoRa communication gateway. From April 24th to May 1st, 20-25 datasets were collected from 8:00 to 17:00 every day, and the collected thermal data and images were stored in the onboard SD card of the host controller.

Using Visual studio 2019 as the platform, the point cloud library PCL1.10.0 and the computer vision library OpenCV3.1.0 (Open Source Computer Vision Library) were installed, and the C++ language for software programming was used to realize data processing.

### 2.3 Data processing

#### 2.3.1 Stereo-calibration of thermal and binocular cameras

Photogrammetry-based registration of thermal and binocular cameras requires stereo-calibration of the two cameras.

**Figure 3:**
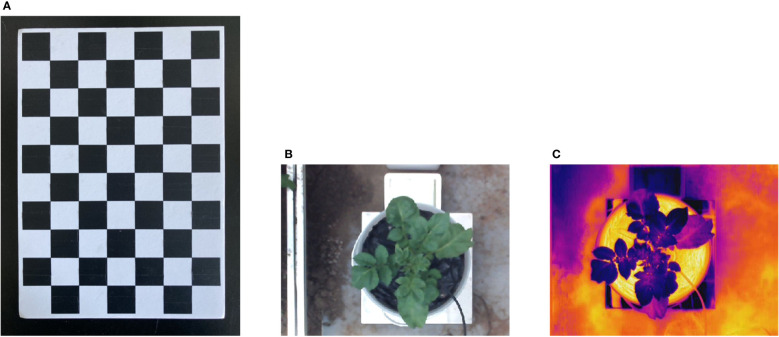
Images pre-processing and Checkerboard. **(A)** Checkerboard for stereo-calibration, **(B)** thermal image of the potato plant, **(C)** cropped and resized color image of the potato plant.

The checkerboard grid (Gan et al., 2018), was used in the experiment for stereo-calibration. The size of each square is 30 × 30mm, as shown in **Figure 3A**. The resolutions and filed of views of the color and thermal images are different. First, the checkerboard in the color image was resized to be the same as that in the thermal image by applying the bicubic interpolation algorithm, and then the color image was cropped to the same resolution as the thermal image to facilitate subsequent registration. The results are shown in **Figures 3B, C**. Next, stereo-calibration was implemented using the Stereo Camera Calibrator toolbox in MATLAB 2018a. The stereo-calibration gets two sets of parameters, the first set of parameters are the elements of interior orientation of the cameras (Wolf et al., 2014). The second set of parameters is named relative orientation between cameras (between the left and right cameras of the binocular camera; between the left color and the thermal cameras) (Gan et al., 2018). The calibrated parameters were saved for subsequent use. Finally, the interior orientation and relative orientation parameters of the left and right cameras of the binocular camera were loaded and the stereo rectification was applied, so that the left and right color images were aligned in parallel without distortion.

#### 2.3.2 Coarse registration of thermal and color images

**Figure 4:**
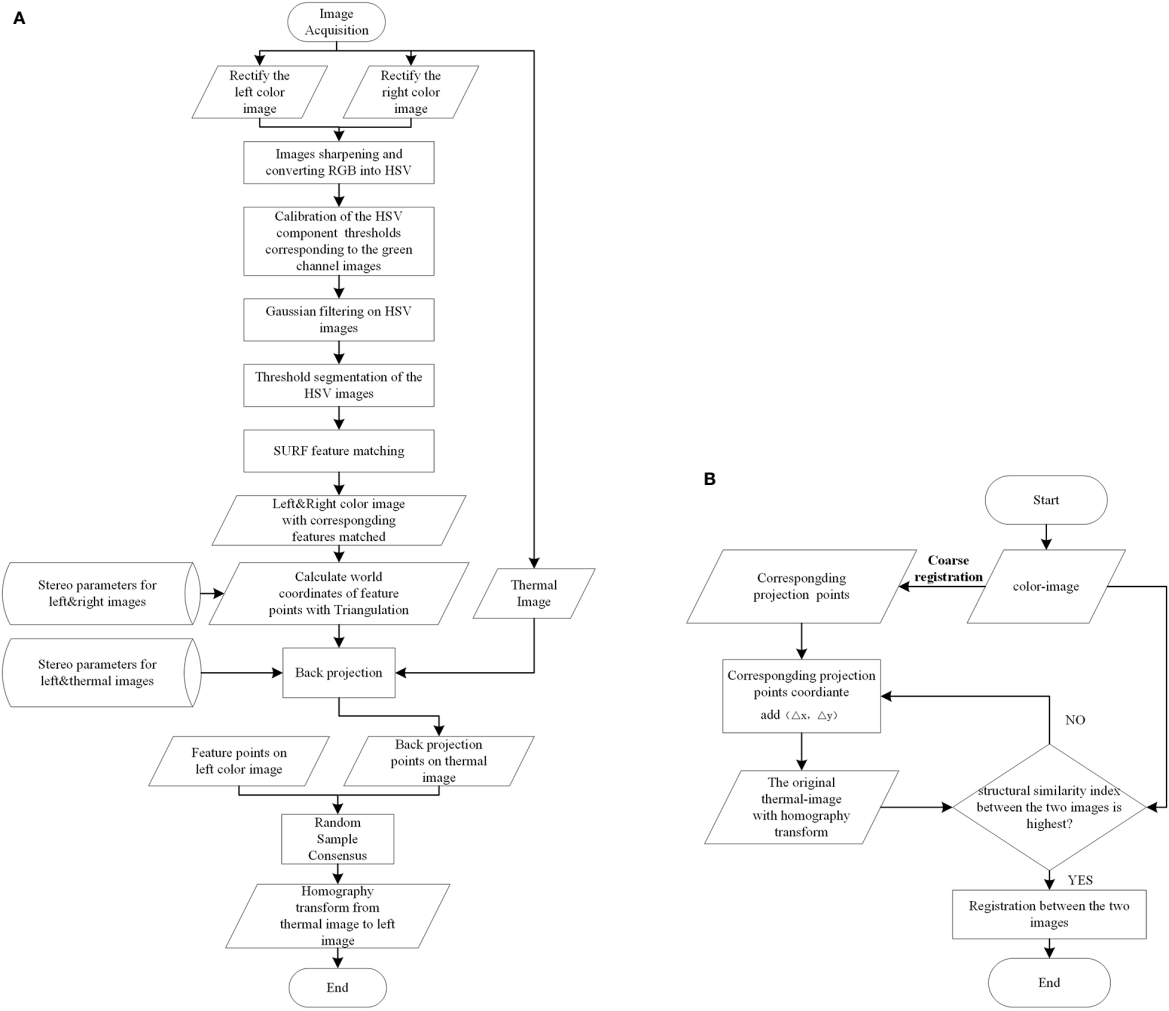
Workflow of the **(A)** coarse registration method and **(B)** fine registration method.

**Figure 5:**
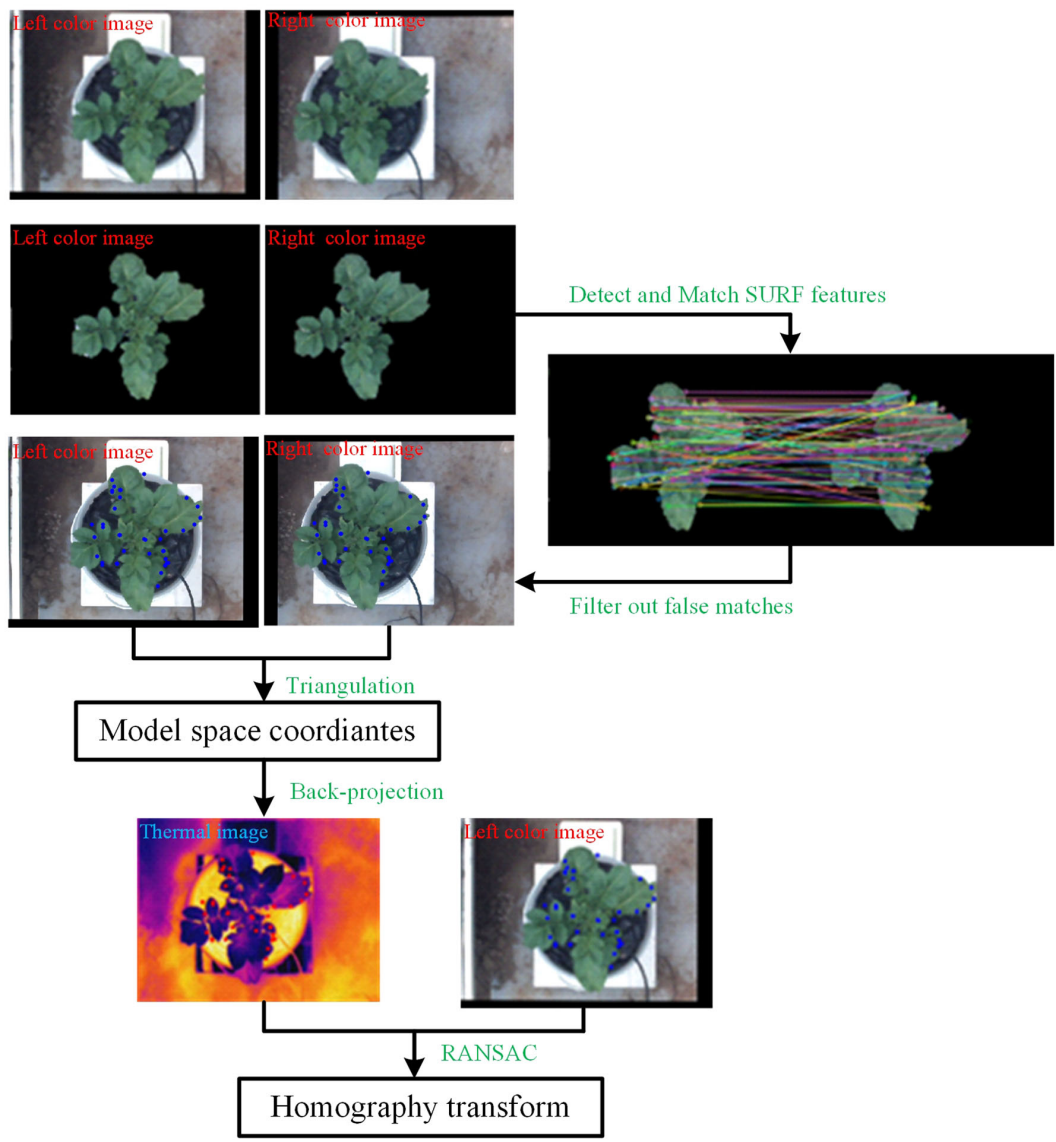
Example of coarse registration of color and thermal images.

The registration between thermal and color images requires finding the geometric transformation relationship between them. This requires groups of homonymy points to be found correctly in two images. The process is shown in **Figure 4A**, and **Figure 5** shows a specific example.

First, to quickly select the homonymy points as many as possible on the potato plants, the Laplacian algorithm was used to sharpen the left and right color images to enhance the contours/edges of the image (Ma et al., 2014). Then the sharpened images were converted into the HSV (Hue, Saturation, Value) color space. Compared with the RGB (Red, Green, Blue) model, the HSV model can express the brightness, hue, and saturation of the color very intuitively, and can effectively use the color space for segmentation (Hamuda et al., 2017). The mask parameters were obtained by setting the upper and lower thresholds of the H channel [HL, HU], S channel [SL, SU], and V channel [VL, VU] to extract the target image, which was the green channel image, through the ‘and’ operation (Li et al., 2020).

Second, speeded-up robust features (SURF) features were detected on the two target images and their descriptors were established. Then, the detected feature points were matched using the nearest neighbor distance ratio strategy and the matching results were displayed on the original images. Some mismatched points in the matching results may negatively affect the registration, filtered out using epipolar geometry constraints.

Third, the filtered feature point pairs were extracted, and the interior direction and relative direction parameters of the left and right cameras of the binocular camera were loaded. The triangulation principle was used for these point pairs to calculate the world coordinates. Back-projection was applied using function (2) to project those world coordinates onto the thermal image.

Lastly, taking the feature points in the left color image and the back-projected points on the thermal image as the input, Random Sample Consensus (RANSAC) algorithm was applied to compute the homography transformation that best describes the relationship between these point pairs. The calculation result of the RANSAC algorithm is the optimal solution in the sense of least squares error. The thermal image was transformed using the resulting transformation matrix.


(2) $${\mathrm{S \times Coordinates}}_{\mathrm{thermal}}\mathrm{= K \times}\left[\begin{array}{cc} R & T\\ 0^{T} & 1 \end{array}\right]{\mathrm{\times Coordinates}}_{\mathrm{world}}$$

where, *S* is a non-zero scale factor, which is the z-component of the world coordinates, Coordinates_thermal_ are the homogeneous coordinates of the back-projection points on the thermal image, and Coordinates_world_ are the homogeneous coordinates of the world points. *K*, *R*, and *T* are the intrinsic matrix of the thermal camera, the rotation matrix, and the translation matrix of the thermal camera relative to the left camera of the binocular camera, respectively.

#### 2.3.3 Fine registration of thermal and color images

The premise of applying the coarse registration method to accurately register color and thermal images is that the photographed object is close to the plane. However, the surface structure of the potato plant canopy is complex and the depth varies greatly. Moreover, the error of the coarse registration is relatively large due to the error brought by the camera calibration. Therefore, when the coarse registration cannot meet the registration accuracy requirements, further fine registration is required. The specific process is shown in **Figure 4B**.

Firstly, for any filtered feature point in the color image, its corresponding point in the thermal image can be obtained by applying the coarse registration method. For instance, if the coordinates of a feature point in the color image were (x_0_, y_0_ ), the corresponding projection point coordinates in the thermal image were calculated by back-projection as (x_0_pro_thermal_, y_0_pro_thermal_) . Secondly, it was assumed that (x_0_pro_thermal_, y_0_pro_thermal_ ) and the coordinates of the true corresponding points in the thermal image (x_0_thermal_, y_0_thermal_ ) had a small position difference (*∆*_x_, *∆*_y_) , which had an initial value (0, 0 ). Thirdly, the filtered feature points in the color image and the position-compensated corresponding points in the thermal image (x_0_pro_thermal_+*∆*_x_, y_0_pro_thermal_+*∆*_y_ ) were retaken as input, and the RANSAC algorithm was used to calculate the homography matrix that can best describe the transformation relationship between them, and the original thermal image was transformed. Fourthly, the structural similarity (SSIM) index was used to measure the similarities between the transformed thermal image and the color image. The two images were first constrained to co-aligned regions by cropping and then used as input for measurement (Dandrifosse et al., 2021). Lastly, repeated the previous four steps by increasing the values (*∆*_x_, *∆*_y_ ). Because the SSIM possesses the property of maximum uniqueness, the transformation with the largest SSIM value was chosen as the optimal transformation. The temperature matrix was acquired simultaneously with the thermal image was also transformed using the optimal homography transformation for subsequent use.

#### 2.3.4 Generation of potato plant PCD with temperature

3D reconstruction based on stereo vision technology is one of the most commonly used reconstruction methods. Stereo matching technology based on image information to acquire depth information is a popular research topic in stereo vision. It is the process of finding the homonymy points in two images, then calculating the disparity value to acquire the depth information of the point in the three-dimensional space.

In this study, stereo vision technology was used to reconstruct the canopies of potato plants in 3D to generate point clouds. Based on the similar triangle principle, the depth of the world coordinate point can be calculated by the following function:


(3) $$\text{D = }\frac{\text{B }∙\text{ f}}{{\text{x}}_{\text{l}}\;{\text{- x}}_{\text{r}}}$$

where *D* is the depth value. *B* is the baseline, which is the distance between the principal points of the two cameras of the binocular camera. *f* is equal to the focal length multiplied by a coefficient representing the number of pixels per millimeter on the imaging plane. d=x_l_-x_r_ is called disparity, which is the difference between the *x* coordinates of the two corresponding pixels on the left and right images.

First, a variant of the semi-global matching algorithm (SGM), the semi-global block matching algorithm (SGBM) was applied, using the left and right color images with stereo rectification in section 2.3.1 to calculate the disparity map. Next, due to occlusion or uneven illumination, some disparity values in the disparity map are unreliable, and median filtering was used to filter out isolated noise points caused by mismatching. After removing false matches, the removed pixels will cause holes of invalid values, and the method of multi-level mean filtering was used to fill the voids iteratively. Multi-level mean filtering is a variant of mean filtering, which is an algorithm that fills holes multiple times by changing the filter window size and using the integral map of the disparity map. It first performs mean filtering with a larger initial window and assigns values to the holes in a large area. Then in the subsequent filtering, the window size was reduced to half of the original size, and the original integral graph was used to filter again and assign values to the smaller holes (overwrite the original values). These steps were repeated until the window size became 3× 3, then the filtering stopped and the final result was obtained. Then, the similar triangle principle was applied using function (3) to calculate the depth value of a point in space, and the three-dimensional coordinate information of the point was calculated in combination with function (4) (Huang et al., 2020; Xie et al., 2020). Finally, based on the homography transformation between the color and thermal images in section 2.3.3, a new point cloud data type was defined by using the PCL library, which integrated the three-dimensional coordinate information, RGB color value, and temperature information of the potato plant canopy together. Thus, the original 3D points cloud data of the potato canopy containing both color information and temperature information has been generated.


(4) $$\{\begin{array}{c} \text{Z =D}\\ \text{X = }\frac{{\text{x - x}}_{0}}{\text{f}}\cdot \text{ D}\\ \text{Y = }\frac{{\text{y - y}}_{0}}{\text{f}}\cdot \text{ D} \end{array}$$

where (x, y) are the pixel coordinates of the image, and (x_0_, y_0_) are the pixel coordinates of the principal point.

#### 2.3.5 Optimization of PCD and extraction of 3D distribution of CWSI

In section 2.3.4, the original PCD of the potato plant were acquired through stereo vision technology. The original PCD not only contained potato plant but also background point clouds such as soil and flower pots. The quality of PCD is not high due to the influence of environmental factors (e.g. illumination, wind speed) and image registration errors. Some methods need to be taken to optimize the original PCD to extract the potato plant canopy information. The specific steps are as follows.

Step one: the PCD of the canopy of the potato plant were extracted. Color is one of the most important features for distinguishing crops and backgrounds in a greenhouse environment (Tkalcic and Tasic, 2003). Philipp and Rath (2002) found that the HSV color space is one of the most reliable color spaces for distinguishing plant from the background. In order to extract the green canopy area of potato plant, a color model based on the HSV color space was used to segment the original PCD.

Step two: the scatter points were removed. The produced scatter points due to factors such as random measurement error and external environment when acquiring PCD was removed by using the statistical filtering algorithm.

Step three: the abnormal temperature points of the canopy were removed. Some ground areas were incorrectly matched to some of the leaves due to the registration errors of the thermal and color images, resulting in higher temperature values o4f the leaves than their true surface temperatures. To remove such incorrectly matched points, the k-means algorithm was used to classify all points into two classes according to their temperature, and the class with more points was saved as the optimized PCD of the potato plant canopy (Qiu et al., 2021).

Step four: the CWSI value of each point of the potato plant canopy was calculated and its 3D distribution was acquired. The temperature of the wet reference surface was acquired from the original PCD, and the temperature of the dry reference surface was replaced by the air temperature plus 7 °C. The CWSI of each point in the canopy can be obtained with these data through function (1) and the distributions of CWSI in 3D were obtained.

### 2.4 Performance evaluation

Evaluation of registration performance was conducted for thermal and color images. Because the true coordinates of the matching points on the thermal image corresponding to the color image cannot be determined, the accurate matching error between these point pairs cannot be calculated. However, the thermal image after the optimal homography transformation can be overlaid with the color image to show the performance of the registration. At the same time, the homography transformation errors between the matching feature points on the color image and transformed thermal image were calculated. Second, registration performance was also measured by computing the average distance between control points (control point error) on the color and thermal images (Dandrifosse et al., 2021). The control points were visually selected by a human operator on the potato plant. The points had to be selected on recognizable pixels (all locations of the canopy and leaves).

## 3 Results

### 3.1 Feature detection and matching results of the left and right color images

**Figure 6:**
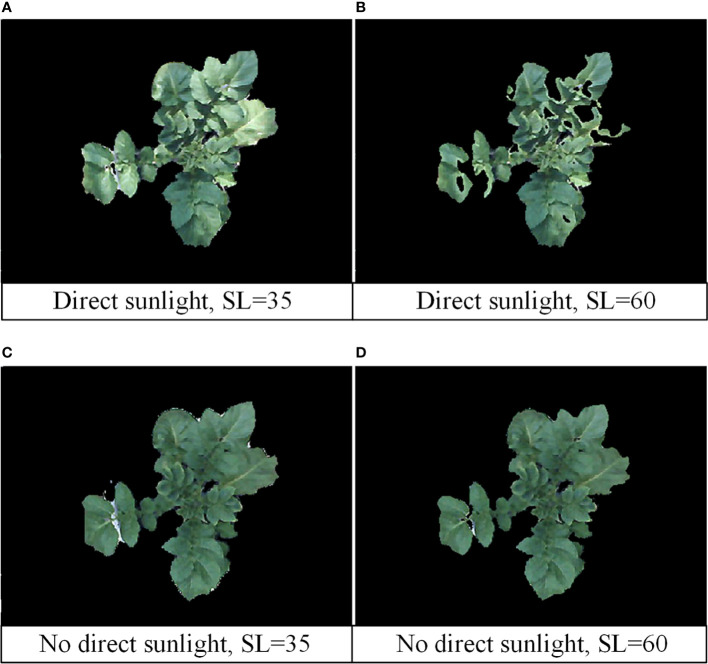
Image processing results under different sunlight and SL thresholds. **(A)** Direct sunlight, SL=35; **(B)** direct sunlight, SL=60; **(C)** no direct sunlight, SL=35; **(D)** no direct sunlight, SL=60.

The image processing shows that illumination affect the specific settings of H, S, V thresholds, especially in the saturation, that is, the parameter S. When the sunlight hits the crop surface directly, it affects the color and brightness of the image, and the SL of the green reference color varies from 30 to 40. The SL of the green reference color varies from 45 to 65 when there is no direct sunlight. The images obtained at different times of the day were analyzed and compared, as shown in **Figure 6**. **Figures 6A, B** show the images when the sunlight directly on the surface of tomato plants. At this time, the effect is best when the SL is 35. **Figures 6C, D** show images of tomato plants in shadow or without direct sunlight. At this point, the best result is when the SL is 60. In the experiment, in order to reduce the interference of background such as soil, a black plastic was covered on the soil surface (**Figure 1A**). Under these conditions, the values of HU and HL were set to 100 and 35, respectively.

**Figure 7:**
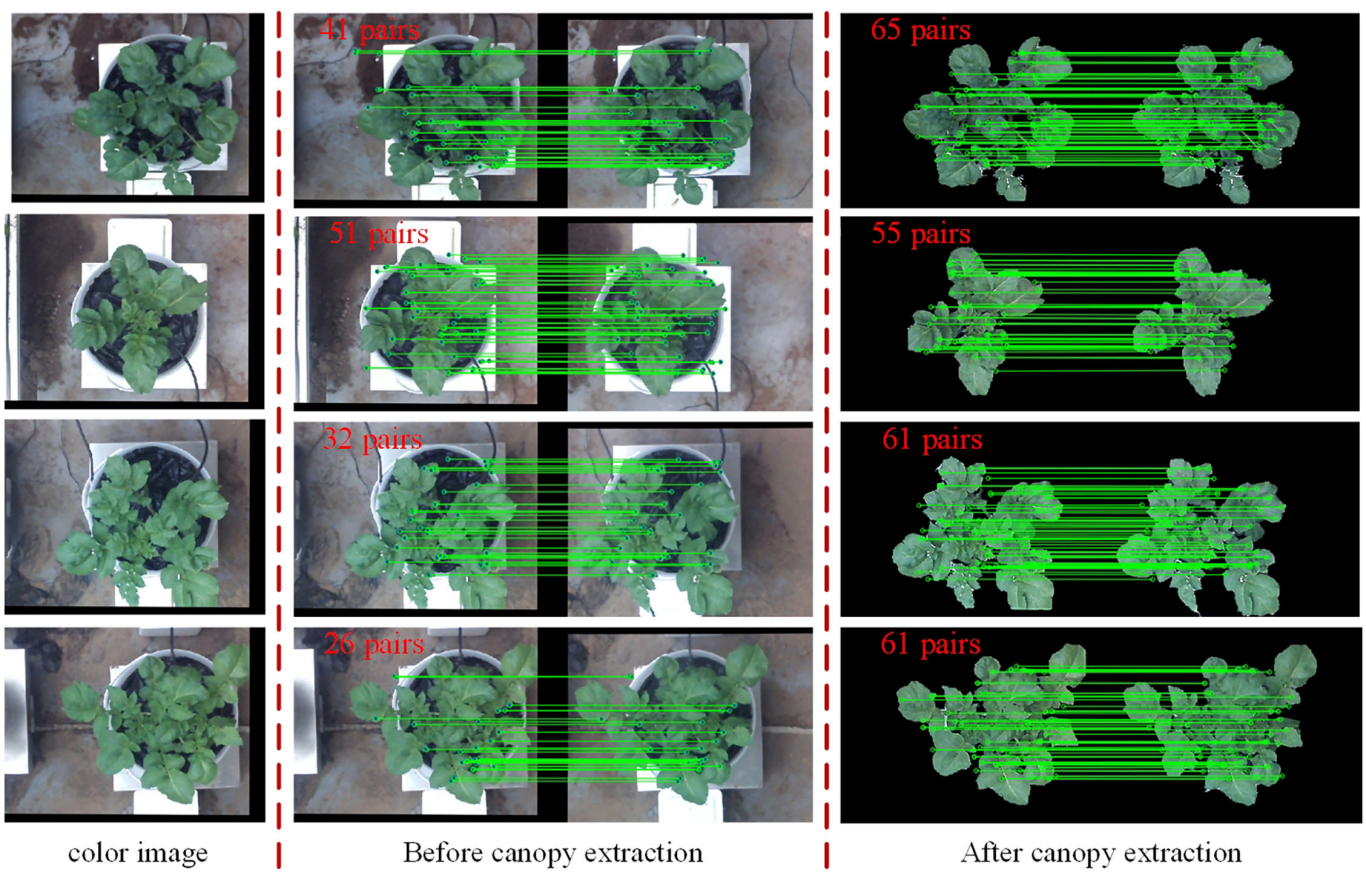
Comparison of the number of feature points obtained by detection, matching and filtering before and after canopy extraction.

The feature detection results on the green channel image were compared with the feature detection results on the original color image. The number of good matching points acquired by filtering before and after extracting the canopy was used to characterize the comparison results (the feature detection, matching and filtering algorithms and parameter settings used before and after the canopy extraction remain the same), as shown in **Figure 7**. In this study, a total of 30 potato plant samples from different collection periods were randomly selected for verification, the results are shown in **Table 1**. It can be seen that the number of matching point pairs filtered from the extracted canopy images is 48% more than the number of point pairs filtered from the original images on average.

**Table 1:** Comparison of the results of feature detection before and after canopy extraction in ten samples.

Sample No.	Amounts of feature points		
	Before canopy extraction	After canopy extraction	Increase (%)
1	41	65	58.5
2	51	55	7.8
3	32	61	90.6
4	26	61	134.6
5	20	34	70
6	33	54	63.6
7	42	68	61.9
8	10	28	180
9	71	78	9.9
10	26	35	34.6
11	48	68	41.7
12	60	81	35.0
13	42	63	50.0
14	47	79	68.1
15	24	60	150.0
16	35	71	100.0
17	41	52	26.8
18	28	65	132.1
19	34	67	97.1
20	38	65	71.1
21	28	42	50.0
22	21	40	90.5
23	46	38	-17.4
24	29	35	66.7
25	66	81	22.7
26	47	66	40.4
27	34	49	44.1
28	75	89	18.7
29	31	42	35.5
30	51	49	-3.9
average	39.2	58.0	48.0

### 3.2 Results of registration for thermal and color images

**Figure 8:**
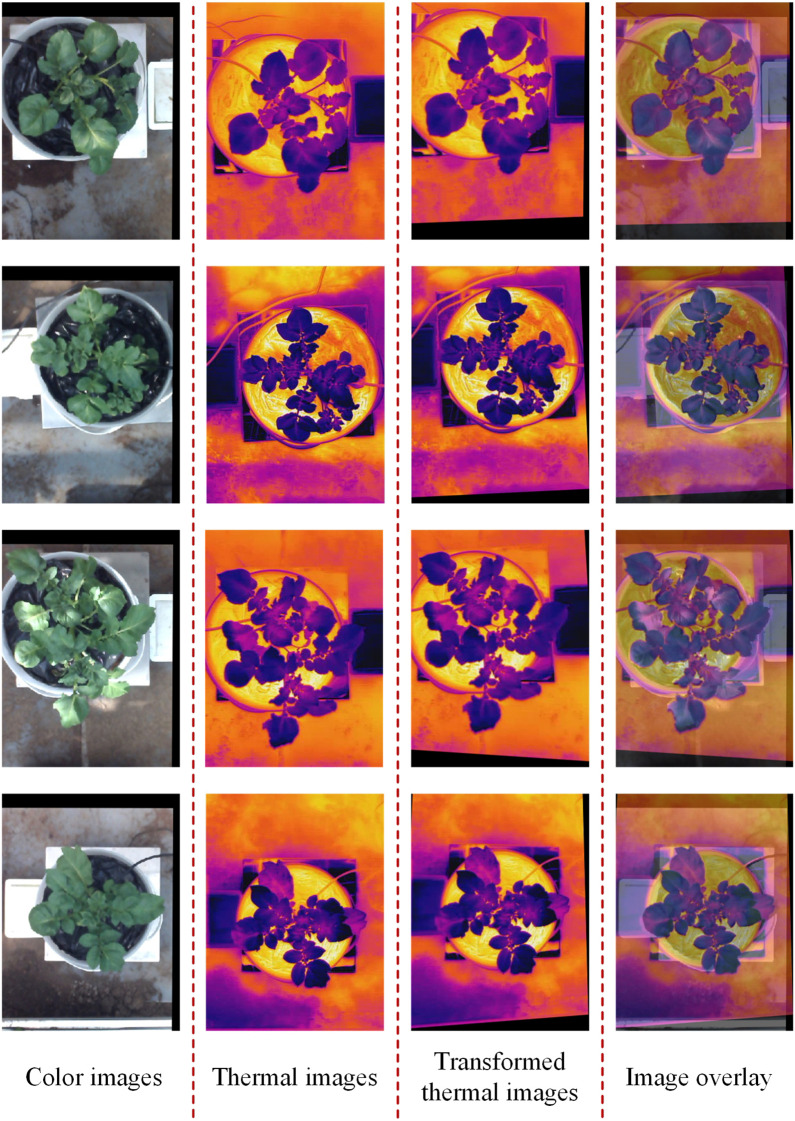
Registration results of the thermal and color images of potato plant.

The registration of thermal and color images was finished according to the procedures described in sections 2.3.2 and 2.3.3. The example in **Figure 8** shows the performance of the proposed image registration method and it can be seen that even though some potato plants have a complex canopy structure and a wide range of depths, their color and thermal images can be well registered. Image registration performance was evaluated with the same set of randomly selected 30 potato plant samples. The homography transformation error and control point error of each potato plant sample was recorded. The statistical results are shown in **Tables 2** and **3**. The registered control point error is 2.8 pixels on average, indicating that the proposed SURF feature detection on the extracted canopy images and photogrammetry-based methods can effectively register the color and thermal images of potato plants.

**Table 2:** Homography transformation errors between color image and transformed thermal image.

Images No.	1	2	3	4	5	6	7	8	9	10
Error (pixel)	0.4	0.5	1.0	0.3	0.3	0.5	0.3	0.8	0.9	0.5
Images No.	11	12	13	14	15	16	17	18	19	20
Error (pixel)	0.3	0.6	0.7	1.0	0.7	0.5	0.4	0.3	0.6	0.6
Images No.	21	22	23	24	25	26	27	28	29	30
Error (pixel)	0.4	0.5	0.3	0.3	0.4	0.6	0.5	0.5	0.3	0.7

Bold values represent image sequences.

**Table 3:** Control Points Error between color image and transformed thermal image.

Images No.	1	2	3	4	5	6	7	8	9	10
Control Points Error (pixel)	3.3	2.8	3.5	2.1	2.7	1.9	2.0	3.1	3.3	2.2
Images No.	11	12	13	14	15	16	17	18	19	20
Control Points Error (pixel)	1.8	3.3	3.7	3.3	4.2	2.7	4.0	2.6	2.4	1.9
Images No.	21	22	23	24	25	26	27	28	29	30
Control Points Error (pixel)	4.5	2.2	3.5	2.8	3.1	2.6	3.4	2.5	2.2	2.3

Bold values represent image sequences.

### 3.3 Extraction of 3D distribution of potato plant CWSI

#### 3.3.1 Results of generation and optimization of the PCD

When using the disparity map calculated by stereo matching for 3D reconstruction, the quality of the generated PCD is often not high due to the low quality of the disparity map. Therefore, some disparity refines operations can be performed to improve the quality of the disparity map. In this study, the SGBM algorithm used, in addition to its sub-pixel fitting and consistency check and other strategies to refine the disparity map, median filtering and multi-level mean filtering algorithms were also adopted to refine the disparity map further. Experiments show that setting the initial window size to 4× 4 not only ensures that the holes were filled, but also ensures that the image is not over-smoothed.

**Figure 9:**
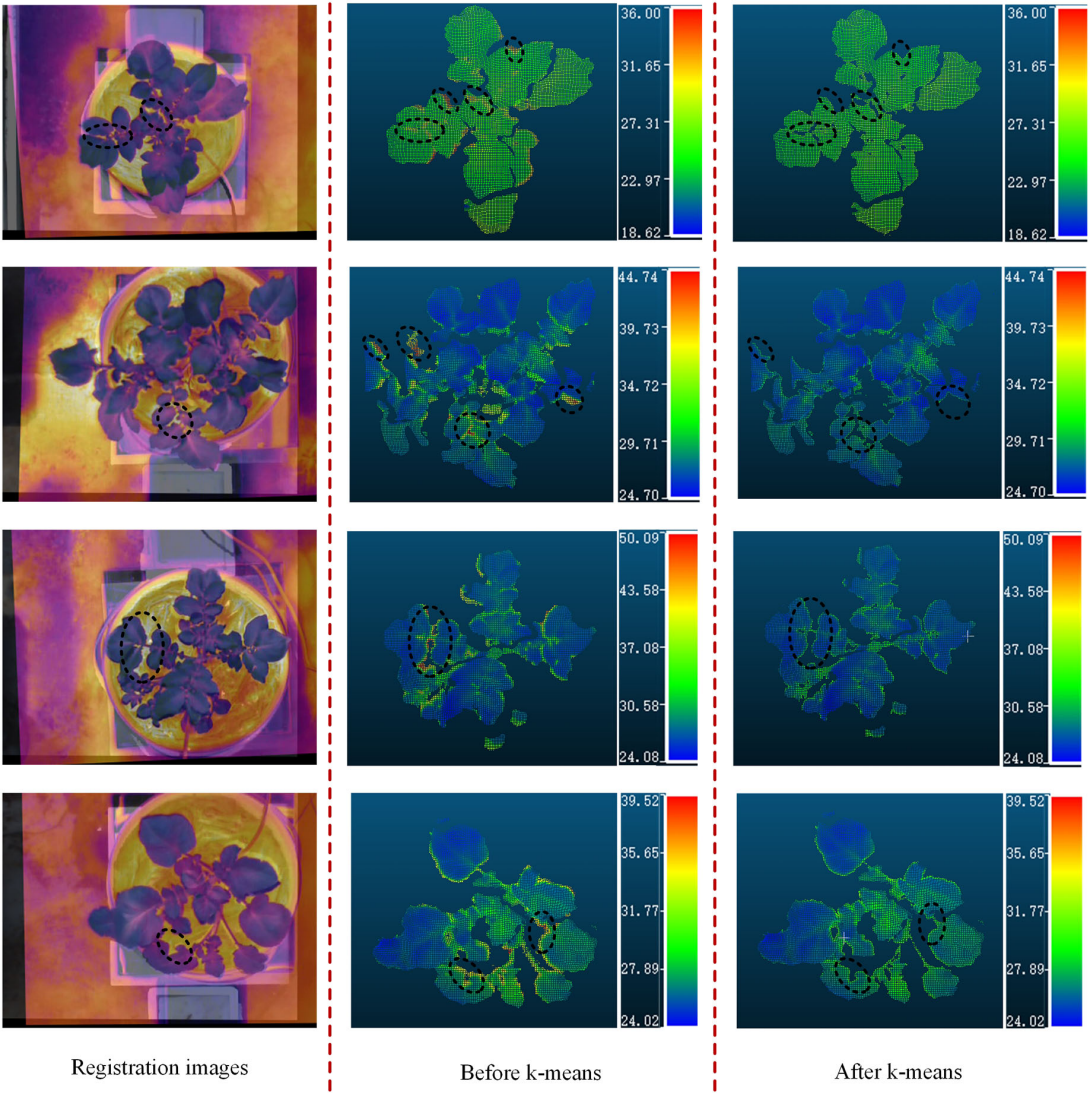
Four examples of the processed PCD of potato plant. PCD of potato plant before the k-means and the PCD of potato after the k-means. (unit: °C).

**Figure 10:**
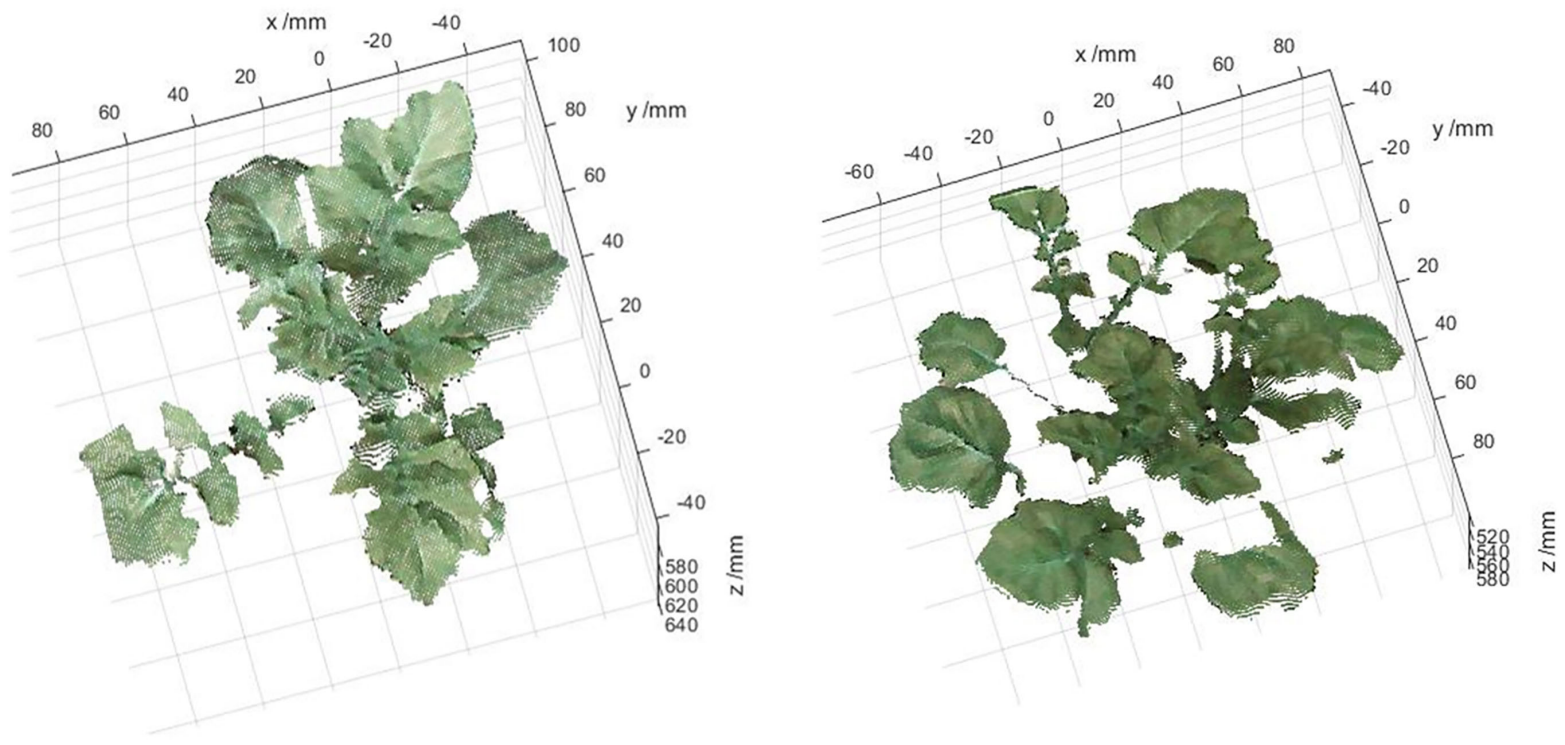
Two examples of the optimized PCD of potato plant. (unit: mm).

The PCD were generated from the stereo-rectified left and right color images and the registration results of the color and thermal images, and contains both color information and temperature information. Firstly, a method based on HSV color space segmentation was used to extract the green canopy of the potato plant, and then only the canopy PCD were operated. However, the resulting PCD also contained many scatter points, which was filtered out by applying a statistical filtering algorithm. In this study, the number of neighbors selected for statistical analysis was 50, and the threshold for identifying outliers was set to 0.5. Besides, the temperature values of some regions were much higher than the real surface temperature of the potato plant, mainly due to the miss matching of the canopy partial PCD and the ground caused by the image registration error. The difference between the temperature of the potato plant and the ground was significant. A clustering algorithm based on k-means was used to filter out abnormal temperature points caused by image registration errors, and the optimized PCD of the potato plant canopy were retained. The PCD of four potato plant samples (two well-watered and two water-stressed) of different sizes and qualities were selected from the treatment and control groups to demonstrate the results of this method. As the black circles shown in **Figure 9**, it can be seen that the temperatures in some edge regions of the canopy were significantly higher than that of other regions before the k-means clustering algorithm was adopted. Through the above treatments, the canopy PCD of the potato plant were relatively intact, as shown in **Figure 10**.

#### 3.3.2 Extraction of plant CWSI in 3D distribution-a case study

In this study, the PCD and temperature data of the potato plant were combined basing color and thermal images registration. The direct approach was used to calculate the CWSI value of each point of the potato plant canopy, and the 3D distributions of the CWSI were obtained.

**Figure 11:**
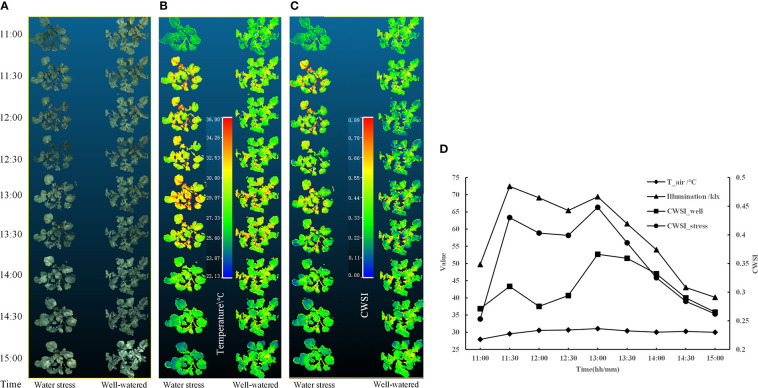
At the conditions of under well-watered and water stress, the distributions of the **(A)** color, **(B)** temperature, **(C)** CWSI of two potato samples in 3D,and **(D)** variation curves of air temperature, illumination intensity, and CWSI of two potato samples.

After extraction of 3D distribution of CWSI, one potato plant sample was selected from each of the data of treatment and control acquired on April 26 (partly cloudy), and the PCD generated from 11:00-15:00 were processed to obtain their distributions of the temperature and 3D CWSI, and the results are shown in **Figures 11A–C**. It can be seen from the figure that the potato petiole and the area around the vein respond quickly to water stress, the temperatures were significantly higher than that of other areas, and the difference under different irrigation treatments was very large, which can provide a reference for the selection of the measurement position of potato plant water stress state. At the same time, the change curve of their average CWSI values of canopy were plotted, as shown in **Figure 11D**. It can be seen from the figure that each two peaks appeared in the CWSI (CWSI_well and CWSI_stress) of the potato plants under two different moisture treatments, and each time appeared at the same time. The first peak appeared at 11:30. At this time, the temperature in the greenhouse was 29.54 °C, which was in the rising stage, and the illumination intensity was the maximum value of 72.4 klx. The second peak appeared at 13:00. At this time, the CWSI of the potato plant under two different water treatments reached the maximum value, the temperature in the greenhouse was 31.05°C, which was the highest value in a day, and the illumination intensity was 69.4 klx. From the above statistical results, it can be seen that the water stress state of the potato plant was jointly affected by air temperature and illumination intensity. The water stress degree of potato plant can be comprehensively evaluated in combination with soil moisture and environmental parameters.

## 4 Discussion

### 4.1 Feature detection and matching of the left and right color images

To quickly find the set of points that best represent the geometric transformation between color and thermal images, and to select these points from the potato plant canopy as much as possible. The RGB color space was converted to HSV color space and extracted the green channel image, by setting the upper and lower thresholds of the H, S, and V values, respectively. The purpose of this is to reduce the interference of background points when computing the homography transformation using the RANSAC algorithm. The results show that the method of feature detection by extracting the canopy provides more and effective candidate matches for computing the optimal homography transformation between color and thermal images.

### 4.2 Registration of thermal and color images

**Figure 12:**
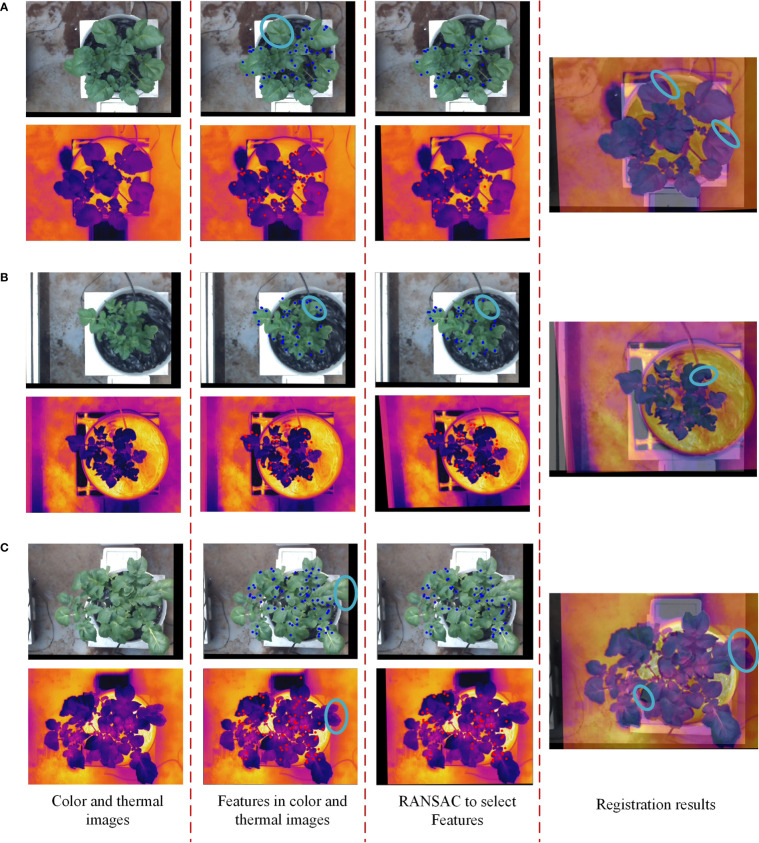
Three examples of potato plants that were not perfectly registered. **(A)** No feature points can be detected in the blue circled area, **(B)** feature points with low intensity in the blue circled region are filtered out, and **(C)** different plant sizes in the blue circled area..

Although the method of extracting the canopy from the color image and then performing the SURF feature detection can provide more candidate matches for image registration, the thermal image after homography transformation sometimes cannot be well registered with the color image. In this study, a similarity index was selected to evaluate the structural similarity between images, and the optimal homography transformation matrix that could ensure more canopy pixel overlap was obtained by finding its maximum value. Nonetheless, some areas in the potato plant canopy were less affected by the homography transformation because no feature points were detected in these areas due to the influence of illumination changes and the weak texture of the canopy surface, as the blue circles shown in **Figure 12A**. It was also because even if feature points were detected in this part of the region, they will finally be filtered out by the RANSAC algorithm due to their low quality, which also caused this part of the region to be less affected by the homography transformation, as the blue circles shown in **Figure 12B**. These situations resulted in some regions cannot be perfectly aligned, increasing the registration error.

As the blue circles shown in **Figure 12C**, it can be seen that some regions in the color and thermal images have large differences in shape, and some edge regions are not absolutely overlapping. One reason for the differences was that the different resolutions of the thermal and color cameras resulted in the cropped color image not being exactly the same size as the potato plant in the thermal image. The other reason was that the thermal and binocular camera were fixed at different positions of the image acquisition module resulting in the potato plant in the captured images having different shape characteristics in some areas. Lastly, although the experiments were conducted in a relatively closed greenhouse environment, ventilation was applied during the day, causing the leaves of the potato plant to swing in the wind sometimes. The differences in the shape of the potato plants in the two images caused by all these factors lead to inevitable registration errors.

### 4.3 Extraction and analysis of 3D distribution of potato plant CWSI

When filtering out abnormal temperature points caused by thermal image and color image registration errors, the k-means algorithm sometimes clustered leaves and soil background points into one class, resulting in false segmentation of some leaves point clouds. This was most likely to occur in the near-ground leaves of potato plants under water stress. The near-ground potato plant leaves under water stress conditions were affected by high-temperature radiation from the soil, and their temperatures were close to or even the same as the ground temperature. When the temperature clustering method based on k-means was applied, this part of the point clouds and the soil point clouds will be clustered into one class, resulting in the false segmentation of canopy point clouds. In this case, the method of accurately extracting the canopy point clouds needs further improvement.

The experimental period of this study was the seedling stage of potato, which was mainly the period for stem and leaf growth and root system development, and the growing amount accounted for about 1/5 of the whole growth period. Most of the leaves in this period were in the early stage of function, and various physiological activities were very vigorous. Therefore, the detection of water stress status in this period can be considered using the entire canopy. However, at the stage of tuber expansion, the growth of shoots and leaves on the ground stopped, and the growth of tuber volume and weight were the main factors. At this time, the potato is most sensitive to water and needs the most water, and the water demand accounts for more than 50% of the water demand in the whole growth period. Therefore, accurate detection of water stress status during this period directly determines tuber size and yield. During this period, the plant canopy size was large, and the leaves at different depths responded differently to water stress due to the difference in chlorophyll content and the length of the functional period. To analyze the water status of potato plant more accurately, some point clouds segmentation techniques to extract a single leaf from the PCD of the potato plant canopy can be considered. A leaf-scale-based water stress status analysis method based on the 3D motion robotic system proposed in this study will be further studied in the future.

## 5 Conclusion

A low-cost 3D motion robotic system for automated high-throughput phenotyping detection of potato plant was developed and demonstrated in this study. The system can continuously acquire potato plant canopy image and thermal data through timing triggering, providing data support for potato plant water status analysis in both space and time scale. The efficiency of data collection using this system was much higher than that done manually.

With the help of this 3D motion robotic system platform, a cost-effective method was proposed to realize the detection of potato plant CWSI in 3D. The green canopy was extracted from a color image of potato plant based on the HSV color space, and the thermal and color images were registered using the SURF algorithm and photogrammetry. The results show that extracting the green canopy from the color image and then performing feature detection can provide more candidate point pairs for computing the homography transformation. The filtered feature points on the color images were projected as world coordinate points, and back-projected onto the thermal image, and then accuracy of these back-projected points was improved to through position-compensated method. Finally, taking the points on the thermal and left color images as input, the optimal homography transformation for each set of images was calculated by the RANSAC algorithm. The average error of the homography transformation was 0.52 pixels, and the average error of the registered control points was 2.8 pixels, indicating that the used method was suitable for registering thermal and color images of potato plants. In addition, the temperature clustering method based on k-means can effectively eliminate the interference of background point clouds. However, for the accurate extraction of point cloud of potato plant canopy under partial water stress, the k-means algorithm needs to be further optimized to improve the segmentation accuracy.

This paper also provided a case study for 3D distribution extraction of CWSI analysis based on the provided 3D motion robotic system. By analyzing the diurnal variation and influencing factors of CWSI, data support can be provided for accurate detection of potato water stress. In the future, the performance of the proposed method will be verified in different growth stages of potato. And the changing of the CWSI 3D distribution in both leaf scale and canopy scale with the continuous changing time under different water stress levels will be studied, which will provide data support for precision irrigation strategy making both in the field and in the greenhouse.

## Data availability statement

The raw data supporting the conclusions of this article will be made available by the authors, without undue reservation.

## Author contributions

LW and HL: Methodology, Software, Writing – original draft, Writing – review and editing. YM: Writing – review and editing. YH, CP: Performed the experimental work, and acquired data. MZ: Supervision, Writing – review and editing.

## Supplementary material

Supplementary Video 1 The workflow of the 3D motion robotic system.
